# The Risk of West Nile Virus Infection Is Associated with Combined Sewer Overflow Streams in Urban Atlanta, Georgia, USA

**DOI:** 10.1289/ehp.1001939

**Published:** 2010-06-08

**Authors:** Gonzalo M. Vazquez-Prokopec, Jodi L. Vanden Eng, Rosmarie Kelly, Daniel G. Mead, Priti Kolhe, James Howgate, Uriel Kitron, Thomas R. Burkot

**Affiliations:** 1 Emory University, Atlanta, Georgia, USA; 2 Centers for Disease Control and Prevention, Atlanta, Georgia, USA; 3 Georgia Division of Public Health, Atlanta, Georgia, USA; 4 University of Georgia, Athens, Georgia, USA; 5 Fulton County Department of Health and Wellness, Atlanta, Georgia, USA; 6 Fogarty International Center, National Institutes of Health, Bethesda, Maryland, USA

**Keywords:** arbovirus, Culex quinquefasciatus, risk factors, spatial clustering, spatial epidemiology, urban pollution

## Abstract

**Background:**

At present, the factors favoring transmission and amplification of West Nile Virus (WNV) within urban environments are poorly understood. In urban Atlanta, Georgia, the highly polluted waters of streams affected by combined sewer overflow (CSO) represent significant habitats for the WNV mosquito vector *Culex quinquefasciatus*. However, their contribution to the risk of WNV infection in humans and birds remains unclear.

**Objectives:**

Our goals were to describe and quantify the spatial distribution of WNV infection in mosquitoes, humans, and corvids, such as blue jays and American crows that are particularly susceptible to WNV infection, and to assess the relationship between WNV infection and proximity to CSO-affected streams in the city of Atlanta, Georgia.

**Materials and methods:**

We applied spatial statistics to human, corvid, and mosquito WNV surveillance data from 2001 through 2007. Multimodel analysis was used to estimate associations of WNV infection in *Cx. quinquefasciatus*, humans, and dead corvids with selected risk factors including distance to CSO streams and catch basins, land cover, median household income, and housing characteristics.

**Results:**

We found that WNV infection in mosquitoes, corvids, and humans was spatially clustered and statistically associated with CSO-affected streams. WNV infection in *Cx. quinquefasciatus* was significantly higher in CSO compared with non-CSO streams, and WNV infection rates among humans and corvids were significantly associated with proximity to CSO-affected streams, the extent of tree cover, and median household income.

**Conclusions:**

Our study strongly suggests that CSO-affected streams are significant sources of *Cx. quinquefasciatus* mosquitoes that may facilitate WNV transmission to humans within urban environments. Our findings may have direct implications for the surveillance and control of WNV in other urban centers that continue to use CSO systems as a waste management practice.

West Nile Virus (WNV) is a mosquito-transmitted virus (family Flaviviridae) and a human, equine, and avian neuropathogen (Kramer et al. 2008). The virus is indigenous to the old world (i.e., Africa and Middle East) and is maintained by a bird–mosquito–bird transmission cycle primarily involving *Culex* sp. mosquitoes; humans, horses, and other mammals are dead-end hosts for the virus (Kramer et al. 2008). Since its emergence in New York City in 1999, WNV has spread over much of North America and the Caribbean and has become a threat to public, equine, and wildlife health (Kramer et al. 2008; Lindsey et al. 2010). Human WNV infections cluster in space and time, with the highest concentrations of cases occurring in urban environments during the summer (Brown et al. 2008; Kramer et al. 2008). Factors that strongly influence WNV transmission dynamics in urban environments throughout the United States include variation in the occurrence and competence of mosquito vectors and vertebrate reservoirs (Kilpatrick et al. 2006a; Reisen et al. 2005, 2008a; Turell et al. 2005), local variation in mosquito feeding patterns (Hamer et al. 2009; Kilpatrick et al. 2006b), bird–herd immunity and infectiousness (Reisen et al. 2008b), WNV genotypes (Bertolotti et al. 2008; Kilpatrick et al. 2008), human population characteristics (Ruiz et al. 2004, 2007), climate, and other environmental variables (Kilpatrick et al. 2008; Soverow et al. 2009).

Information on the distribution and abundance of mosquito vectors in urban environments is paramount for assessing local risks of exposure to WNV. In the United States, members of the *Culex pipiens* complex (*Cx. pipiens* and *Culex quinquefasciatus*), *Culex tarsalis*, *Culex stigmatosoma*, *Culex salinarius*, *Culex nigripalpus*, and *Culex restuans* have been implicated as urban vectors of WNV (Hayes et al. 2005; Reisen et al. 2008a; Turell et al. 2005). Most of these species (particularly the *Cx. pipiens* complex) require organic-rich water for larval development and are commonly found in high numbers in artificial containers, unattended pools, retention ponds, storm drains, catch basins, sewage systems and treatment plants, and pit latrines (Calhoun et al. 2007; Curtis and Feachem 1981; Reisen et al. 2008c).

Combined sewage and street runoff discharges into natural waterways are considered the main sources of urban stream pollution (Paul and Meyer 2001; Tibbetts 2005). According to the U.S. Environmental Protection Agency (EPA), about 850 billion gallons of untreated mixed wastewater and stormwater are discharged annually into U.S. urban waterways, mainly through combined sewer overflow (CSO) systems (Tibbetts 2005; U.S. EPA 2004). In times of low precipitation, CSO systems channel wastewater to a treatment plant before it is discharged into a waterway. However, treatment facilities are by-passed during heavy precipitation or snowmelt, and combined wastewater and stormwater are discharged directly into natural waterways after only minimal chlorine treatment and sieving to remove large physical contaminants. Because of concerns about their potential impacts on both public health and the environment, CSO systems have become the focus of a debate regarding the best techniques to manage growing volumes of sewage and stormwater runoff in many U.S. communities (Tibbetts 2005). About 40 million people in 772 cities from 32 states live in cities with CSO systems; most are located in the mid-Atlantic and midwestern states (U.S. EPA 2004).

An unforeseen effect of CSO discharges into natural streams has been the increased breeding of mosquito species that are considered important vectors of human pathogens. In a study performed in two creeks in the city of Atlanta, Georgia, Calhoun et al. (2007) found that streams that received nutrient pulses from CSO systems had increased numbers of *Cx. quinquefasciatus,* the main vector of WNV in the area. Other studies have shown that a faster mosquito larval development (Curtis and Feachem 1981), coupled with a reduced larval predation and intraspecific and interspecific competition (Agnew et al. 2000; Beketov and Liess 2007; Mogi and Sota 1996), and a significant tendency of females to lay their eggs in organically enriched waters (Chaves et al. 2009) have contributed to the high *Cx. quinquefasciatus* productivity in CSO-affected streams. As *Cx. quinquefasciatus* larval productivity levels in CSO streams seem not to be significantly affected by distance from the source of overflow discharges (Calhoun et al. 2007), the potential impact of such environments as sources of WNV vectors could extend throughout their drainage path, covering a significant proportion of the urban space. However, to our knowledge, an assessment of the potential contribution of CSO-affected streams as sources of WNV infection for humans and birds has never been undertaken.

In this study, our objectives were to describe and quantify the spatial distribution of mosquito abundance and WNV infection in mosquitoes, humans, and birds, specifically corvids (blue jays and American crows), that are considered sentinels of local virus activity (Nielsen and Reisen 2007) in the city of Atlanta, Georgia, from 2001–2007 and to assess the relationship between CSO-affected streams and WNV infection.

## Materials and Methods

### Study area

From October 2001 through December 2007, the state of Georgia reported 199 human WNV infections and 17 deaths [Centers for Disease Control and Prevention (CDC) 2010]. Of these, 25.1% resided in Fulton County, the most populous county in Georgia (992,472 inhabitants based on 2008 census estimates) and the core of the Atlanta metropolitan area. Fulton County covers 1,385 km^2^ and encompasses populations with a wide range of socioeconomic conditions, from the wealthiest neighborhoods in the state to those with the highest poverty rates in the United States. Seven CSO facilities and the streams associated with them (CSO streams) and about 76 non-CSO streams (excluding tributary creeks) are located within Fulton County.

### Data sources and management

Geographic coordinates of each CSO facility and associated streams and data on mosquito abundance and WNV infections in humans, mosquitoes, and bird carcasses from October 2001 through December 2007 were provided by the state of Georgia and the Fulton County Department of Health and Wellness (FCDHW). The protocols for data management, analysis, and dissemination were approved by the CDC Institutional Review Board committee (protocol 990121).

From 21 August 2001 through 31 October 2007, mosquitoes were trapped only in urban environments, and the trapping was concentrated in the city of Atlanta (where most of the population of Fulton County resides). They were collected overnight using paired CO_2_-baited CDC light traps (Silver 2008) and gravid traps (Reiter 1983) that were placed within 5 m of each other at each sampling location. A total of 455 trap locations were sampled in Fulton County [average number of trap locations per year was 93.7 ± 47.8 (± SD)]. Trap locations were clustered in Fulton County but were randomly distributed within the city of Atlanta [see Supplemental Material, Figure 2 (doi:10.1289/ehp.1001939)]. Every year, mosquito sampling was performed biweekly from 1 May through 31 October by FCDHW staff.

Mosquitoes were classified by species; pooled by date, trap location, species, and trap type; and tested for WNV infection [average number of mosquitoes tested in each group was 13.4 (range, 1–25); see Supplemental Material (doi:10.1289/ehp.1001939) for details on the WNV testing procedures]. To derive WNV infection rates, we used the maximum likelihood (ML) estimation tool (Biggerstaff 2006) to account for variation in the numbers of mosquitoes tested (WNV-positive mosquitoes per 1,000 tested). The intensity of WNV infection was defined as the average ML WNV mosquito infection rate for the period 2001–2007.

We used geocoded data on the 50 human cases of WNV reported in Fulton County from 2001–2007 and human population data from the 2000 U.S. census (U.S. Census Bureau 2000) to estimate annual human WNV infection incidence rates (cases/100,000 persons) for each census tract. Incidence rate estimates for relatively uncommon (or underreported) diseases such as WNV can be imprecise, particularly for small administrative units such as census tracts (Waller and Gotway 2004). Thus, we used spatial Empirical Bayes (EB) smoothing methods that reduce variance by borrowing information from neighboring spatial units. However, because there is a tradeoff between improved precision and the introduction of bias due to the smoothing process (Waller and Gotway 2004), we have reported the results of spatial analyses based on both smoothed (EB) and unsmoothed human WNV incidence rates.

The locations of WNV-positive bird carcasses submitted by citizens to health authorities were geocoded (ArcGis 9.2; ESRI, Redlands, CA). We used data on WNV-positive corvid (i.e., blue jays and American crows) carcasses only, as a consequence of their high WNV mortality and proximity to humans (Nielsen and Reisen 2007). We divided the number of WNV-positive corvid carcasses per census tract by the 2000 population estimate (U.S. Census Bureau 2000) to obtain the number of WNV-positive corvids per 100,000 persons per year in each census tract (i.e., referred to as the WNV-positive corvid death ratio). This metric allowed minimizing bias due to nonrandom variation in the submission of bird carcasses across tracts. We have reported the average WNV-positive corvid death ratios for each tract from August 2001 through December 2004 because bird carcass submissions were highest during this time period.

Environmental factors that might influence mosquito spatial distribution and WNV infection were derived from various data sources. Elevation (in meters above sea level) was derived from the Shuttle Radar Topography Mission (SRTM) data set (90-m pixel size and pixel values describing elevation), generated by the Consultative Group on International Agricultural Research-Consortium for Spatial Information (CGIAR-CSI 2008). Distances (in meters) to the nearest CSO stream and the nearest catch basin (potential *Cx. quinquefasciatus* urban larval habitats) were estimated by a straight line function in ArcGis 9.2 (expressed as a raster layer with a 30-m pixel size and pixel values indicating the distance of the pixel to the nearest CSO stream or catch basin. The extent (percentage) and variability (range) in tree canopy cover and the percentage of land covered by different land-use classes were obtained from the National Land Cover Database (30-m pixel size and pixel values indicating either percentage of canopy cover or land-cover type) generated from Lansat satellite images collected in 2001 by the Multi-Resolution Land Characteristics Consortium (MRLC 2008).

Predictors of *Cx. quinquefasciatus* abundance and WNV infection rates (distance to CSO and catch basins, elevation, land use) were estimated at each trap location by averaging values within 1 km of each location (to account for spatial dependence in trap collections due to mosquito flight). Tree canopy range was estimated by calculating the range of canopy cover values within a 1-km radius of each trap. Predictors of human WNV incidence rates and WNV-positive corvid death ratios were estimated at the census tract level. Environmental factors (distance to CSO and catch basins, elevation, land use, and canopy coverage) were averaged per tract, whereas tract estimates of sociodemographic factors (i.e., median household income and percentage of houses built in the 1950s and 1960s) were obtained from the 2000 U.S. census (U.S. Census Bureau 2000). Vegetation, elevation, household income, and age of the houses were identified as significant predictors of WNV human infection in the Greater Chicago area (Ruiz et al. 2004).

### Statistical analyses

We used spatial statistics to identify the location and extent of statistically significant clustering (*p* < 0.05) of high *Cx. quinquefasciatus* abundance and WNV infection in humans and corvids [see Supplemental Material (doi:10.1289/ehp.1001939) for a detailed description of the statistics].

The spatial distribution of *Cx. quinquefasciatus* abundance was mapped using a Kernel density function (Silverman 1986) and expressed as mosquitoes per hectare. We used Getis’ G_i_*(*d*) statistic (Ord and Getis 1995) to detect spatial clusters of high *Cx. quinquefasciatus* abundance per trapping location (the 2001–2007 average number of *Cx. quinquefasciatus* per trap-night). Significant clustering of *Cx. quinquefasciatus* WNV infection intensity per trap location (the 2001–2007 average ML WNV infection rate) was also tested by G_i_*(*d*). We used the Bernoulli spatial scan test (Kulldorff 1997) to detect the presence of significant clustering of *Cx. quinquefasciatus* WNV infection (the presence or absence of WNV infection on a trapping location during 2001–2007). A local Moran’s I local indicator of spatial association (LISA) test (Waller and Gotway 2004) was used to identify significant clustering of human WNV infection rates and WNV-positive corvid death ratios. Statistical inference was based on the difference between observed values and expected values generated from 999 Monte Carlo randomizations.

We assessed relationships of *Cx. quinquefasciatus* abundance and WNV infection intensity, human EB WNV incidence rates, and WNV-positive corvid death ratios with selected environmental and demographic characteristics (including the average distance to CSO streams and catch basins, land use, percentage of tree cover, median household income, and housing characteristics) using a multimodel selection approach (Burnham and Anderson 2002). Under this analytic framework, we contrasted a set of candidate models (representing different hypotheses) with each other and identified the best model (or the best set of models) based on model fit (Burnham and Anderson 2002), where the best model was the one with the lowest Akaike information criterion (AIC) value. When the lowest AIC value differed from the next best model by < 2 units, we identified a best set of models rather than a single best model (Burnham and Anderson 2002). We estimated the Akaike weight (ω*_i_*) for each model as a measure of the probability that a particular model fitted the data better than the alternative set of candidate models (Burnham and Anderson 2002). For each independent variable (*j*) evaluated, we estimated the sum of Akaike weights (∑ω_i_) as the sum of the ω*_i_* from each model in which *j* was a statistically significant predictor of the outcome (Burnham and Anderson 2002). This metric (bounded between 0 and 1) evaluates the relative importance of each independent variable for predicting the dependent variable (Burnham and Anderson 2002). We contrasted seven models (a full model and six linear combinations of environmental, demographic, and spatial factors) for *Cx. quinquefasciatus* abundance and WNV infection and five models for human WNV incidence rates and for WNV-positive corvid death ratios.

We performed the statistical analyses using STATA (version 9.0; StataCorp, College Station, TX); for all spatial analyses, we used ClusterSeer2 (TerraSeer, Ann Arbor, MI), GeoDa (http://geodacenter.asu.edu/) and SatScan (http://www.satscan.org/).

## Results

Only data from *Cx. quinquefasciatus* collected from gravid traps (38,120 of the total 71,824 mosquitoes collected) were used in this study [see Supplemental Material (doi:10.1289/ehp.1001939)]. Yearly averages of *Cx. quinquefasciatus* ML WNV infection rates varied between 2.6 and 16.3 per 1,000 mosquitoes, with no clear temporal pattern (Table 1). Data were available for 321 WNV-positive corvids, which accounted for 98.2% of the WNV-infected dead birds collected in 2001–2006 [see Supplemental Material, Table 1 (doi:10.1289/ehp.1001939)]. The declining temporal trend in WNV-positive corvid death ratios (from 28.0 per 100,000 persons in 2002 to 0.00 per 100,000 in 2006; Table 1) is most likely explained by the decreasing number of dead birds submitted by Atlanta residents to the FCDHW. Of the 50 confirmed cases among Fulton Country residents in 2001–2007, 26 (52%) had symptoms of neuroinvasive WNV. The 2001–2007 average WNV incidence rate among Fulton County residents (0.8 cases per 100,000) was two times higher than the 1999–2008 national average (0.4 cases per 100,000) (Lindsey et al. 2010). With the exception of the years 2001 and 2006, human incidence rates showed little temporal variation, ranging between 0.93 and 1.0 cases per 100,000 (Table 1).

**Figure 1:**
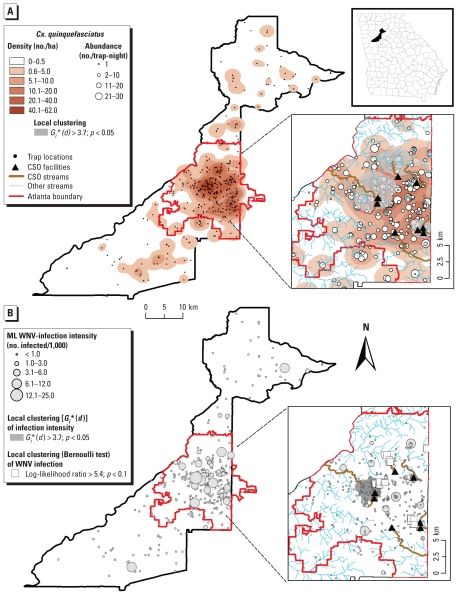
(*A*) Abundance (mosquitoes/trap-night), density distribution (mosquitoes/ha), and local spatial clustering of *Cx. quinquefasciatus* abundance, 2001–2007. (*B*) Distribution of WNV infections in *Cx. quinquefasciatus* and location of clusters of high ML WNV infection intensity [G_i_*(*d*)] and presence (Bernoulli test) in mosquito pools (*Cx. quinquefasciatus* grouped according to date and location) tested during 2001–2007. Large insets show a detailed view of the city of Atlanta, whereas the upper right inset shows the location of Fulton County.

*Cx. quinquefasciatus* was heterogeneously distributed, with the highest abundances (number per trap-night) and densities (numbers per hectare) concentrated within the boundaries of the city of Atlanta (Figure 1A). Traps with elevated abundance of *Cx. quinquefasciatus* mosquitoes clustered in three locations within an average radius of 1,305 m (range, 200–2,000) from each trap (Figure 1A) [G_i_*(*d*) > 3.7; *p* < 0.05]. The three high mosquito abundance clusters encompassed segments of five of the seven CSO streams in Atlanta (Figure 1A). High WNV infection intensity in *Cx. quinquefasciatus* (WNV-positive mosquitoes/1,000) also clustered in three locations within Atlanta (Figure 1B, G_i_*(*d*) > 3.7; *p* < 0.05). The largest cluster [average radius = 1,042 m (range, 400–2,000)] surrounded part of the North Avenue CSO creek and encompassed 41 trapping locations (Figure 1B). The remaining two clusters encompassed only two trap locations each, with clustering radii of 320 and 93 m, respectively (Figure 1B). Clusters of WNV infection presence in *Cx. quinquefasciatus* (presence/absence of WNV infection at a trapping location) also occurred within the city of Atlanta, in proximity [average = 988 m (range, 10–3,118)] to CSO streams (Figure 1B) (Kulldorff Bernoulli test, *p* < 0.05). K-function analysis (Ripley 1976) confirmed that the observed clustering patterns were not the result of heterogeneous trap distribution, because traps were randomly distributed within the city of Atlanta [Supplemental Material, Figure 2B (doi:10.1289/ehp.1001939)].

The proportion of WNV-infected *Cx. quinquefasciatus* pools (mosquitoes grouped by date and location) was significantly higher within 1 km of a CSO stream (125/1,215) than within 1 km of a non-CSO stream (77/1,044) (*p* = 0.027). Furthermore, 9 of 12 (75%) trapping locations where WNV-infected *Cx. quinquefasciatus* mosquitoes were detected during early spring were located within 1 km of a CSO stream. The pattern of ML WNV infection clustering in *Cx. quinquefasciatus* was consistent across years, with most positive pools located in close proximity to CSO streams [see Supplemental Material, Figure 3 (doi:10.1289/ehp.1001939)].

A regression model that included the average distance between each trapping location to the nearest CSO stream and the range of tree canopy cover within 1 km of each trap location was best supported by the data (ω*_i_*, 65.8%) (Table 2). Distance to CSO streams was the best predictor of the abundance of *Cx. quinquefasciatus* mosquitoes (∑ω*_i_* = 100%, by being statistically significant in the best models), followed by tree canopy coverage range (∑ω*_i_* = 66.9%) (Table 2). The remaining environmental factors were not statistically significant. Traps with a high density of *Cx. quinquefasciatus* (> 20/trap-night) were located at an average distance of 1,156 m (95% CI, 552–1,759 m) from a CSO stream and had an average tree canopy range of 98% (95% CI, 95–99%). Tree canopy range can be interpreted as the degree of canopy variability around a trap location, as high values include highly forested and deforested areas close to each other (within 1 km).

The presence of WNV infection in *Cx. quinquefasciatus* was also predicted by the average distance to CSO streams and by the canopy cover range (Table 2). The two best candidate models included distance to CSO streams as the sole significant predictor of *Cx. quinquefasciatus* WNV infection presence. Distance to CSO streams (∑ω*_i_*, 80.2%) followed by canopy cover range (∑ω*_i_*, 15.2%) were the most important predictors in these models.

**Figure 2:**
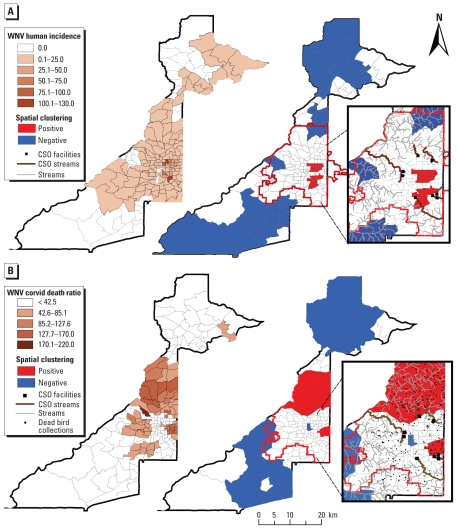
Distribution and spatial clustering of (*A*) EB-smoothed WNV human incidence rate estimates (cases/100,000 persons) and (*B*) WNV-positive corvid death ratios (number of dead corvids/100,000 persons) in Fulton County. Inset shows a detailed view of the city of Atlanta.

Human WNV incidence rates (EB) and WNV-positive corvid death ratios also clustered within the city of Atlanta (Figure 2). One of the two clusters of high human WNV incidence (identified by the Local Moran’s I LISA test) was located near three of the seven CSO streams (Figure 2A). This cluster included six census tracts and six reported cases and had an average annual incidence (± SD) of 48.6 ± 37.4 cases per 100,000 persons. The second (northern) cluster of human WNV incidence was not directly associated with any CSO stream (Figure 2A). This cluster included 10 tracts and 10 cases and had an average annual incidence of 42.6 ± 31.3 cases per 100,000 persons. In contrast, Atlanta census tracts outside the clustering areas had an average annual incidence of 7.5 ± 11.0 human WNV cases per 100,000 persons. Clustering zones were similar but encompassed fewer tracts when we repeated the analysis using unsmoothed (versus EB) human WNV incidence rates [see Supplemental Material, Figure 4 (doi:10.1289/ehp.1001939)]. Spatial clustering of WNV-positive corvid death ratios overlapped with human WNV incidence clusters and *Cx. quinquefasciatus* infection clusters in the east and north of Atlanta (Figure 2B). Four of the seven CSO streams were partly encompassed by WNV-positive dead corvid ratio clusters (Figure 2B).

The human WNV incidence rate per census tract (2001–2007, log_10_ + 1 transformed EB estimates) was best predicted by a model that included the average distance from each tract to the nearest CSO stream, mean tree canopy cover, the proportion of houses built in the 1950s–1960s, median household income, and number of WNV-positive dead corvids identified during 2001–2004 (Table 3) (*R*^2^ = 0.21; *p* < 0.001; ω*_i_* = 81.7%). When all variables with *p* < 0.07 were considered, distance to CSO streams was the best predictor of human WNV incidence (∑ω*_i_* = 100%), followed by the proportion of houses built in the 1950s–1960s (∑ω*_i_* = 94.0%) and the median household income (∑ω*_i_* = 92.0%) (Table 3). The remaining factors were poorly supported by the data. Similarly, log_10_ + 1 transformed WNV-positive corvid death ratios were best predicted by a model including the average distance to the nearest CSO stream, mean tree canopy cover, proportion of houses built in the 1950s–1960s, and median household income (Table 3) (*R*^2^ = 0.31; *p* < 0.001; ω*_i_* = 91.3%). Again, mean distance to CSO streams and mean tree cover (both ∑ω*_i_* = 100%), followed by median household income (∑ω*_i_* = 91.0%), were the best predictors of high WNV-positive corvid death ratios (Table 3).

## Discussion

The effect of sewage-affected waterways on mosquito dynamics and the pathogens they transmit have been the focus of research in urban environments throughout the world (Curtis and Feachem 1981; El Esnawy and Nagwa 2001; Mogi and Okazawa 1990), but have received limited attention in the United States, even though approximately 746 urban centers are affected by CSOs (U.S. EPA 2004). Surprisingly, the emergence of WNV and other mosquito-transmitted pathogens as a consequence of combined sewer systems is not considered in the 2004 report of the U.S. EPA as a potential human health impact of CSO systems (U.S. EPA 2004). Previous research in the city of Atlanta, Georgia, showed that CSO-affected streams have higher *Cx. quinquefasciatus* populations and enhance oviposition of female *Cx. quinquefasciatus* compared with non-CSO streams, where *Cx. quinquefasciatus* is rarely found (Calhoun et al. 2007; Chaves et al. 2009). Our study builds on that knowledge and shows that WNV infection in *Cx. quinquefasciatus* is significantly higher in CSO streams than in non-CSO streams and that WNV incidence rates in both humans and corvids are significantly associated with proximity to CSO-affected streams.

According to the U.S. EPA, there is limited information about the human health impacts of CSO systems (Tibbetts 2005; U.S. EPA 2004). Most of the available data focus on the effects of CSO-associated exposures to microbial pathogens (e.g., bacteria, virus, and parasites), toxics (e.g., heavy metals), and biologically active chemicals (e.g., hormones) on human health (Tibbetts 2005; U.S. EPA 2004). Our study shows that WNV infection is significantly higher in close proximity to CSO-affected streams. Similarly, proximity to a sewage plant and its effluent was identified as the only predictor of high WNV human seroprevalence in the city of Hashimiah, Jordan (Batieha et al. 2000). Assuming a *Cx. quinquefasciatus* urban flight range of approximately 1.0 km (Reisen et al. 1991), the potential influence of CSO streams as sources of WNV infected vectors could extend over significant portions of the urban space. In Atlanta, the area potentially affected by flight-dispersed mosquitoes originating from CSO streams could encompass 23% (78.3/341.1 km^2^) of the city. These findings may have direct implications for U.S. urban centers that still rely on CSO systems as a primary waste management practice, particularly those located in mid-Atlantic and midwestern states affected by WNV epidemics (Brown et al. 2008).

Spatial clustering of WNV infection in urban environments occurs in areas where favorable larval breeding conditions, competent reservoir hosts, and opportunities for human exposure overlap (Hamer et al. 2009; Nielsen et al. 2008; Ruiz et al. 2004). In Atlanta, such conditions occurred in close proximity to CSO streams that also were characterized by a larger proportion and range of tree canopy cover, low median incomes, and a large proportion of houses built in the 1950s–1960s. Urban tree cover could have facilitated contact between *Cx. quinquefasciatus* and an abundant bird population (Hamer et al. 2009) and mosquito dispersal away from CSO streams. Because we averaged collections across years, our measures of *Cx. quinquefasciatus* clustering cannot be considered surrogates of mosquito dispersal. Rather, we consider such distances as measures of the extent of high abundance of *Cx. quinquefasciatus* populations. On the other hand, people residing in low-income 1950s–1960s housing areas may be at increased risk of exposure to evening-biting *Cx. quinquefasciatus* compared with residents of high-income areas where air conditioning and protective behaviors such as the use of mosquito repellent or the active avoidance of mosquitoes may be higher. The CSO streams located in the north of Atlanta (one of the wealthiest sectors of the city) overlapped with clusters of WNV infection in mosquitoes and corvids but not human infections, supporting the potential for differential exposure of the human population in such areas. Conversely, in southeast Atlanta (a mid- to low-income area) clustering of WNV infection in humans, corvids, and mosquitoes overlapped. Located within 2.5 km of two CSO streams and connected to them by forest corridors, this area includes a cemetery, recreational areas (e.g., Grant Park, Atlanta Zoo), and commercial and residential spaces, and it has an estimated human WNV incidence rate that is 6.5 times higher than the Atlanta average. Understanding sociodemographic and environmental determinants of virus amplification and transmission dynamics in such transmission hot spots may help explain WNV infection persistence and circulation within urban environments (Bertolotti et al. 2008; Hamer et al. 2009; Ruiz et al. 2004).

Human WNV infection is considered endemic in Atlanta, but the mechanisms explaining WNV persistence in this area are still unclear. The overwintering of the virus in hibernating mosquitoes has been proposed as a mechanism of WNV persistence (Reisen et al. 2006). In the present study, WNV-infected *Cx. quinquefasciatus* mosquitoes identified in early spring were located in close proximity to CSO streams and facilities, which may have been a consequence of the greater abundance of mosquitoes in CSO versus non-CSO streams. In addition, CSO tunnels and exit pipes may serve as ideal mosquito (and virus) overwintering habitats. Our recent finding of a WNV-infected *Cx. quinquefasciatus* pool inside CSO tunnels (Vazquez-Prokopec GM, unpublished data) supports such a hypothesis.

In 1999, the city of Atlanta was found to be in violation of both the federal Clean Water Act and the Georgia Water Quality Control Act because of CSO discharges into urban waterways (U.S. EPA 1999). As a part of a major settlement between the city and U.S. EPA, an underground reservoir system to hold excess effluent during heavy precipitation was constructed (at an estimated cost of $3.9 billion) and completed in 2008. The new CSO system was designed to minimize pollution by reducing the number of overflows to < 10. However, this system could provide an ideal environment for *Cx. quinquefasciatus* mosquitoes and WNV amplification in CSO streams: infrequent flooding with high organic content effluent into streams, a food-rich and predator-free larval habitat, and abundant forest corridors and avian hosts. Further monitoring of mosquito populations in CSO streams in Atlanta is needed to determine if the remediation system will enhance the growth of mosquito populations and further increase the risk of WNV transmission to humans.

## Conclusions

We conducted an integrated analysis of information on WNV infections in mosquitoes, dead corvids, and humans and found a strong association between WNV infection and proximity to CSO-affected urban streams. Our findings are consistent with previous research indicating that CSO streams represent major mosquito-breeding habitats and support the need to expand this research to other urban centers that still include CSO systems as part of their wastewater management strategy.

## Figures and Tables

**Table 1:** WNV infection in *Cx. quinquefasciatus* mosquitoes, corvid carcasses, and humans, Fulton County, Georgia, USA, 2001–2007.

	*Cx. quinquefasciatus*		Corvids		Humans	
Year	No. tested pools (no. individuals)	Average WNV infection rate^a^ (range between traps)	WNV-positive corvids (birds tested)	WNV-positive corvid death ratio^b^	WNV cases	WNV incidence rate^b^
2001	216 (1,968)	16.3 (11.3–22.9)	49 (77)	5.8	1	0.118
2002	749 (12,885)	4.5 (3.4–5.8)	242 (309)	28.0	8	0.927
2003	256 (2,907)	4.6 (2.6–7.7)	19 (59)	2.2	9	1.020
2004	490 (7,267)	9.0 (7.0–11.5)	10 (35)	1.1	9	0.994
2005	750 (8,716)	4.3 (3.0–5.8)	1 (25)	0.1	9	0.963
2006	247 (2,611)	7.2 (4.4–11.1)	0 (11)	0.0	4	0.415
2007	267 (3,090)	2.6 (1.2–5.0)	0 (0)	0.0	10	1.008

a The number of infected mosquitoes per 1,000 tested, estimated using ML methods.

b WNV-positive carcasses per 100,000 humans (WNV-positive corvid death ratios) or human cases per 100,000 (human WNV incidence rates). We used population estimates for every year (U.S. Census Bureau 2000) in the estimation.

**Table 2:** Summary of multiple logistic regression models used to evaluate the average abundance of *Cx. quinquefasciatus* (mosquitoes/trap-night) and WNV infection presence (presence/absence of WNV-positive pools in a trapping location), urban Atlanta, Georgia, USA, 2001–2007.^a^

					Residential use										
Response variable, model^b^	Distance to CSO (m)	Distance to catch basin (m)	Tree cover range (%)	Mean tree cover (%)	Low (%)	Medium (%)	High (%)	Wetland (%)	Forest (%)	Barren land (%)	Elevation (m)	Constant	AIC	ΔAIC	ω*_i_*^c^
*Cx. quinquefasciatus* abundance															
1	−1.2E–4*	—	0.08*	—	—	—	—	—	—	—	—	1.67	2999.3	0.0	0.658
2	−1.2E–4*	—	—	—	—	—	—	—	—	—	—	9.00*	3001.6	2.3	0.208
3	−1.4E–4*	5.0E–4	—	—	—	—	—	—	—	—	—	8.99*	3002.7	3.4	0.120
4	−1.3E–4*	04.3E–4	0.11*	0.02	4.10	−4.63	0.88	1.33	8.28	79.32	0.03	1.79	3007.5	8.2	0.01
5	—	—	—	—	6.35	2.00	5.97	0.17	0.22	90.82	−0.02	3.68	3012.2	12.9	0.01
6	—	—	0.12*	0.05*	—	—	—	—	—	—	—	−1.23	3012.2	12.9	0.01
7	—	4.8E–4	—	—	—	—	—	—	—	—	—	8.32*	3015.5	16.2	0.0
∑ω*_i_*	1.000	0.0	0.669	0.01	0.0	0.0	0.0	0.0	0.0	0.0	0.0				

WNV infection in *Cx. quinquefasciatus*															
1	6.9E–4*	—	—	—	—	—	—	—	—	—	—	−1.26*	406.2	0.0	0.559
2	7.5E–4*	2.4E–4	—	—	—	—	—	—	—	—	—	−1.28*	407.83	1.63	0.247
3	—	—	0.04*	0.04	—	—	—	—	—	—	—	−3.70	408.8	2.6	0.152
4	—	—	—	—	1.40	−4.44	0.04	−3.33	3.33	22.19	−0.03	−1.88	412.15	5.95	0.029
5	2.1E–5	1.3E–4	0.04	1.31	−1.23	1.55	6.51	6.95	−4.07	21.23	−0.01	−4.03	413.9	7.7	0.012
6	—	3.5E–4	—	—	—	—	—	—	—	—	—	−1.51*	419.67	13.47	0.001
∑ω*_i_*	8.1E–1	0.0	0.152	0.0	0.0	0.0	0.0	0.0	0.0	0.0	0.0				

a Results show the parameter estimates and significance for each factor; different models are ordered from best to worst.

b Each candidate model had 455 observations. Observations were based on estimates performed within 1 km of a mosquito trap location. Dashes within the cells indicate that the factor was not included in the model; numbers represent the parameter estimate for each factor.

c Akaike weights, ω*_i_* *= exp*(−1/2 ΔAIC) / ∑*exp*((−1/2 ΔAIC).

* *p* < 0.05.

**Table 3:** Summary of linear regression models evaluated for human WNV incidence rates and WNV-positive corvid death ratios per census tract in Atlanta, 2001–2007.^a^

					2000 U.S. Census						
Response variable Model^b^	Mean distance to CSO (m)	Mean distance to catch basin (m)	Mean tree cover (%)	Mean elevation (m)	Percent houses 1950s–1960s	Median household income (U.S. dollars)	No. dead corvids	Constant	AIC	ΔAIC	ω*_i_*^c^
Human WNV incidence											
1	−6E–5**	—	−0.0018	—	0.0276*	−1E–5*	−0.0362	3.60*	309.0	0.0	0.817
2	−6E–5**	5E–5	−0.0012	−0.0018	0.0287*	−2E–5*	−0.032	4.13*	313.1	4.1	0.105
3	−8E–5*	3E–5	—	—	—	—	−0.117*	2.65*	314.8	5.8	0.04
4	—	—	—	—	0.0381*	−2E–5*	—	3.58*	316.8	7.8	0.02
5	—	—	−0.020*	−0.0035	—	—	—	3.75*	316.9	7.9	0.02
∑ω*_i_*	9.7E–1^c^	0.0	0.02	0.0	0.94	9.2E–1	0.04				

WNV-positive corvid death ratio											
1	−6E–4*	—	0.194*	—	0.1095	1.0E–5*	—	4.26*	707.0	0	0.913
2	−6E–4*	2E–5	0.215*	−0.0159	0.1023	−6.0E–5	—	10.46*	711.7	4.7	0.09
3	—	—	—	—	0.186*	1.4E–4*	—	1.19	728.2	21.2	0.00
4	—	—	0.172*	−0.0017	—	—	—	4.83	728.4	21.4	0.00
5	−3E–4	−1E–5	—	—	—	—	—	11.61*	739.9	32.9	0.00
∑ω*_i_*	1.0	0.0	1.0	0.0	0.0	9.1E–1					

a Results show the parameter estimates and *p*-values for each predictor; different models are ordered from best to worst.

b Each candidate model included 455 observations. Numbers indicate the parameter estimate for each variable included in a given model. Dependent variables (human WNV incidence rates and WNV-positive corvid death ratios) were log_10_+1 transformed, whereas independent variables were estimated over each census tract.

c Akaike weights, ω*_i_* *= exp*(−*1/2* ΔAIC) / ∑*exp*((−1/2 ΔAIC).

c After including variables with *p* < 0.07 in the estimation of ∑ω*_i_*. Without including marginally significant variables, the sum of Akaike weights drop to 0.04.

* *p* < 0.05;

** *p* < 0.07.
